# Teaching foundational language equity concepts in the pre-clinical curriculum

**DOI:** 10.1186/s12909-024-05474-3

**Published:** 2024-05-02

**Authors:** Maria Gabriela Valle Coto, Reniell X. Iñiguez, Marina A. Lentskevich, Syeda Akila Ally, Julia F. Farfan, Yoon Soo Park, Ananya G. Gangopadhyaya, Pilar Ortega

**Affiliations:** 1 Yale New Haven Hospital, New Haven, CT USA; 2 McGaw Medical Center of Northwestern University, Chicago, IL USA; 3 Department of Medical Education, University of Illinois College of Medicine, 808 S. Wood Street, MC 591, Chicago, IL 606012 USA; 4 Department of Diversity, Equity, and Inclusion, Accreditation Council for Graduate Medical Education, Chicago, IL USA

**Keywords:** Language equity, Undergraduate medical education, Language-appropriate care, Health equity, Non-English language preference, Limited English proficiency

## Abstract

**Background:**

Despite the prevalence of non-English languages in the US population, existing medical training to teach communication with linguistically diverse communities is limited to electives or solely focuses on medical interpreting. Language-appropriate communication skills are seldom comprehensively integrated in medical education. This study describes the development and evaluation of an intervention to teach foundational language equity concepts.

**Methods:**

The authors implemented a pre-clinical language equity course at three medical school campuses between August 2020 and March 2022. Sessions focused on the impact of language in health, physician language proficiency standards, and working with medical interpreters. The study sought to (1) understand students’ language skills and prior clinical experiences with patients with non-English language preference and (2) evaluate the curriculum’s impact. Students self-reported their language skills and experiences as part of a voluntary pre-questionnaire. Pre and post-questionnaires evaluated knowledge, attitudes, and intent to apply language equity concepts. Descriptive statistics and chi-squared tests were used to examine trends; themes were identified from free-text responses.

**Results:**

Overall, 301 students completed the course, 252 (83%) completed at least one questionnaire; for each session, between 35% and 46% of learners completed both pre and post-questionnaires. Three quarters (189/252) reported non-English languages. Over half (138/252) reported previous non-English language patient care, and 28% (62/224) had served as ad hoc (untrained) interpreters. Only two students (< 1%) had ever been assessed for medical language abilities. Students demonstrated improved post-course language equity knowledge, strategies for interpreter-mediated encounters, and likelihood to report a plan for language skills assessment (all *p* < .001). Most plans were multifaceted (61%, 38/62), involving goals like completing a language course, taking a proficiency exam, openly discussing skills and uncertainties with team members, and increasing professional interpreter utilization.

**Conclusions:**

A longitudinal language equity curriculum can be feasibly integrated in pre-clinical education, highlight the linguistic diversity of the student body, and serve as a first step in ensuring that all students have a strong language equity foundation prior to clinical rotations. Future steps include evaluating the intervention’s potential long-term effects on professional interpreter utilization, student clinical performance, and institutional culture that promotes multilingualism.

## Background

Over 67 million individuals in the United States speak a non-English language at home [1]. At least 38% of these individuals speak English less than very well and are labeled as having limited English proficiency (LEP) [2]. Recently, a more inclusive term, non-English language preference (NELP), has emerged to describe individuals who can best communicate in a language other than English with respect to a particular type of service or encounter, such as medical care [3]. When individuals with NELP seek healthcare, language discordance between clinicians and patients often leads to suboptimal communication and poor outcomes [4] Language discordance can be successfully addressed either through a professional interpreter (who does not eliminate the language discordance but serves as a mediator for effective communication between clinician and patient) [5] or by matching the patient with a language-concordant clinician (thereby achieving language-concordant care) [6]. While mandates from the federal government require meaningful healthcare access in a patient’s preferred language [7], implementation and utilization of language services (e.g., medical interpreters) and confirmation of non-English skills for clinical use vary significantly across states and healthcare systems [8, 9]. 

Data show that hospitals frequently underutilize professional interpreters [10–13] and medical students and physicians use their non-English skills in patient care even when they recognize their skills are limited [10, 14]. A recent risk assessment study evaluated why physicians choose to ‘get by’ with limited language abilities and identified “lack of physician knowledge and skills” related to language-appropriate communication strategies, such as working with interpreters, as the most common factor and the most amenable to intervention [15].

To improve clinicians’ skills in language-appropriate care, some prior work has explored medical education curricula related to language equity. A 2017 US survey by Himmelstein et al. gathered responses from a quarter of US allopathic medical schools and found that 29 out of 38 schools “provided specific instruction addressing how to work with medical interpreters and/or patients with LEP [16].” The rest of the survey focused on training related to working with interpreters and did not address provision of language-concordant care or student recognition of their own language skills/limitations. Himmelstein et al. point out that “a few schools reported having a large bilingual student population and therefore did not see the need for this instruction” – an alarming statement that illustrates how institutions may view language-appropriate care as the responsibility of some but not all clinicians. In stark contrast to the hundreds of hours dedicated to English clinical skills education, institutions commonly check the box on teaching language-appropriate care by categorizing *all* non-dominant linguistic groups (of which more than 350 exist in the US) as LEP and providing a one-time educational intervention. Even clinicians with full proficiency in one or more non-English languages are likely to encounter patients with whom they are language-discordant.

Some published curricula [3, 17–22] have sought to increase physician trainee knowledge on how to work with professional interpreters. Unfortunately, data demonstrate that students and clinicians persistently underutilize medical interpreters and use their own language skills to “get by” in taking care of linguistically minoritized patients.10 Hence, it is not enough to teach student *how* to work with interpreters; they also need to know *when* to do so – a clinical decision-making skill that should factor in the clinician’s language skills and limitations, the medical complexity and urgency of the situation, and management of limited resources (e.g., requesting an onsite interpreter versus using a telephonic interpreter).

Medical language courses (e.g., medical Spanish) aim to improve language concordance for direct patient-clinician communication and teach learners to recognize their limitations in the target language [23]. However, these opportunities are typically offered as electives, focus on one language, and require a minimum language proficiency level; [24] as a result, they are only accessible to a small subset of trainees. Additionally, multilingual students or physicians may feel overburdened by frequent requests to serve as ad hoc interpreters themselves [14]. Efforts to improve language-appropriate healthcare through medical education should target all students rather than only those eligible for a language course, and should do more than teach students how to work with interpreters. A comprehensive and nuanced approach to language equity is needed to prepare future physicians for effective communication with all patients.

To address current gaps, we developed and evaluated an intervention to longitudinally integrate foundational language equity concepts within the required pre-clinical curriculum at three medical school campuses.

## Methods

The University of Illinois College of Medicine (UICOM) has historically been home to one of the most diverse student populations in the mainland US [25] and has three campuses: Chicago, Peoria, and Rockford each in an urban, suburban, and rural setting, respectively. UICOM’s required Doctoring and Clinical Skills (DoCS) longitudinal course spans the 18-month pre-clinical curriculum.

### Educational intervention

We developed three language equity sessions to be imparted within the DoCS curriculum at all three UICOM campuses. To develop each session, we created a coalition comprised of a faculty member with content expertise in language-appropriate healthcare, a DoCS faculty member, and several medical students. The group was linguistically diverse, representing eight non-English languages (Belarusian, Bengali, Hindi, Polish, Russian, Spanish, Ukrainian, and Urdu). The coalition met monthly between August 2020 and March 2022 to refine the content, learning activities, and questionnaires for each session.

We reviewed conceptual frameworks in the literature, seeking approaches that would allow us to best address the identified gaps in language equity education [26], and ultimately selected two: First, self-determination theory, proposes that addressing student competence, autonomy, and relatedness improves learning, and has been applied in medical education through small group activities and case-based learning [27]. Understanding how to build linguistic competence, graduated autonomy in language-concordant care, and the interdependence/intersection of clinician skills and patient language preference are aspects of language equity that have not been formally addressed through prior curricula and can be explored through a self-determination lens. Second, community cultural wealth [28] is a framework based on social capital theory [29] that posits that individuals’ cultural—including linguistic—assets can be sources of power and social mobility. Medical students’ pre-existing language skills intersect with their early childhood lived experiences, personal and familial national origin, ethnicity, and cultural heritage. If acknowledged and promoted, this linguistic capital can not only enrich the classroom but also healthcare interactions with patients.

Drawing on these conceptual frameworks and based on previously identified gaps in physician knowledge [15] and structural barriers to language-appropriate care [30], each session (Table 1) focused on one of three main areas: (1) the role and impact of language in health, (2) physician language proficiency standards for clinical use, and (3) working effectively with medical interpreters. One faculty member led all three sessions, and the coalition recruited and trained faculty, residents, and senior medical students with prior experience caring for NELP patients to serve as small group facilitators. Each session involved two hours of pre-session student preparation, consisting of three or four articles and a 90-minute live virtual meeting with several interactive components (detailed in Table 1).

**Table 1:** Session learning objectives and activities for the three-part longitudinal language equity curriculum

Session	Learning Objectives Following the session, students will be able to	Learning Activities
**1. Role and impact of language in health**	• Describe the linguistic diversity of the US population and its intersectionality with marginalized identities • Understand what is meant by language-appropriate healthcare and its relation to health equity • Analyze ethical issues related to language-appropriate care	• Pre-readings [6, 31, 32] • Didactics led by medical educator • Video role play of a clinical scenario with a non-English speaking patient • Facilitator-led small group case discussion • Large group debriefing
**2. Physician language proficiency standards**	• Identify validated tools for physician language assessment • Analyze how physicians at different language proficiency levels can appropriately use language skills in a clinical context • Reflect on their own non-English language skills, as applicable	• Pre-readings [15, 33–35] • Didactics co-led by medical educator and language professor • Individual activity to practice using a validated assessment tool • Facilitator-led small group debriefing about individual activity
**3. Working effectively with medical interpreters**	• Describe the role of professional medical interpreters • Describe the roles and responsibilities of physicians in interpreter-mediated encounters • Propose strategies for working effectively with medical interpreters	• Pre-readings [5, 8, 14] • Didactics led by medical educator • Panel of medical interpreters with moderated Q&A • Video of an interpreter-mediated healthcare encounter where medical errors occur [21, 36] • Facilitator-led small group debriefing to discuss strategies to improve communication in the video • Video showing the same encounter with improved communication [35]

### Participants

All 301 medical students in the class of 2024 at the three medical school campuses (182, 65, and 54 at Chicago, Peoria, and Rockford campuses, respectively) participated in the language equity curriculum and were invited to complete voluntary pre- and post-session questionnaires. Informed consent was obtained from all participants. Pre-questionnaires were sent approximately one week prior to the session, and a reminder and opportunity to complete the survey was provided during the first few minutes of the session. Post-questionnaire links were provided in the final five minutes of the session, and students received an email reminder within one week after the session. The University of Illinois Institutional Review Board approved this study on August 15, 2017 (protocol# 2017 − 0482).

### Questionnaires

Coalition leaders created questionnaires by reviewing the available literature and applying their experience as multilingual clinicians and trainees. We identified one validated tool to classify student language proficiency, the Interagency Language Roundtable scale for healthcare, or ILR-H, a self-reporting tool validated for use by health professionals [37]. Due to the paucity of questionnaires in the literature to gather and track data regarding language use in healthcare, other items were developed using research team member expertise following guidance from Artino et al. [38] Questionnaires were piloted with student members of the coalition prior to implementation.

Pre-session questionnaires asked students to indicate their language skills and prior experiences related to language in healthcare. Specifically, we asked about any prior training or exposure related to medical interpreting and caring for patients with NELP. Items with multiple choice responses allowed for optional free-text responses. For the free-text items that would be used for qualitative analysis, an ethnographic approach was used since the goal was to enable respondents to describe prior experiences relevant to the care of linguistically diverse populations.

Some items aimed to assess changes in learner knowledge and attitudes pertaining to each session’s topic. Knowledge questions were multiple choice or true/false items. Attitudes questions with 4-point Likert scale response options (Strongly Agree to Strongly Disagree) asked about student confidence in several common situations, such as using their non-English language skills for patient care, or recognizing potential communication errors that may occur when providing medical care for patients with NELP. Finally, the post-session questionnaires elicited student intent to apply the concepts learned and gathered feedback, including suggestions for future improvements.

### Statistical analysis

Aggregate data on self-reported race and ethnicity for the class of 2024 were used to inform overall descriptive statistics about the cohort of participants. We used descriptive statistics (frequencies and proportions) to examine trends in questionnaire responses. To evaluate internal structure validity evidence of survey responses, we used Cronbach’s alpha to examine internal-consistency reliability. To evaluate validity evidence supporting relations to other variables, we examined pre-post changes to learners’ responses. More specifically, responses were dichotomized (Agree/Strongly Agree v. Disagree/Strongly Disagree) to facilitate interpretation; learner responses between pre- and post-session changes in knowledge and attitudes were compared using chi-squared tests. We conducted analyses using both paired pre-post data (restricting analysis to learners who completed both pre- and post-questionnaires) and unpaired data (using all data collected); we opted to display the results of both analytical methods to maximize the inclusion of data from all respondents. Data compilation and analyses were conducted using Stata 17 (College Station, TX).

Once data were fully deidentified, we reviewed qualitative responses using Microsoft Excel. Coding and inductive analysis followed the Standards of Reporting Qualitative Research [39]. Two research team members (M.V.C and J.F.) reviewed qualitative responses from selected questionnaire items with free-text response opportunities and identified codes and sub-themes individually for each item. We further analyzed sub-themes and grouped them together in overarching themes.

## Results

Each of the three live language equity sessions were held at the three sites between December 2020 and May 2021. Across the three sites, all 301 students completed the full curriculum, and 83% (252/301) responded to at least one of the questionnaires; paired response rates, indicating students who completed both pre- and post-questionnaires, were 46% (session one), 42% (session two), and 35% (session three). The overall racial/ethnic demographic distribution of students was as follows: 38% White (115/301), 24% Asian (73/301), 15% Hispanic/Latinx (46/301), 12% Black/African American (36/301), < 1% American Indian/Alaska Native (1/301), 5% Multi-race (16/301), and 5% Unknown (14/301). Response rate per site was 96% (175/182), 69% (45/65), and 81% (44/54) at Chicago, Peoria, and Rockford campuses, respectively.

### Descriptive statistics

Overall, 252 (83%, 252/301), 158 (86%, 158/182), and 224 (74%, 224/301) students completed the pre-questionnaires for the first, second, and third sessions, respectively. Following each session, 117 (38%, 117/301), 67 (36%, 67/182), and 79 (26%, 79/301) students completed the post-questionnaires for the first, second, and third sessions, respectively. Internal-consistency reliability (Cronbach’s alpha) of survey items (24 questions with rating responses) was 0.73, demonstrating good reproducibility. As previously noted, we ran all analyses both using unpaired data to maximize use of all student responses, and using paired data that restricted the analyses to respondents who responded to both pre and post-questionnaires. Depending on the session, the paired response rate ranged between 35 and 46%. Overall, while there are modest changes in effect sizes when analyzing paired versus unpaired data, our findings indicated no changes in statistical inference (i.e., statistical significance remained the same across the vast majority of items). Additionally, pre and post-subgroups in the unpaired data did not differ in their linguistic profile (*p* = .097).

Most respondents (75%; 189/252) indicated having skills in a language besides English, with over 38 languages represented. The most common non-English language spoken across all three campuses was Spanish (46%, 116/252), followed by French (8%, 19/252), and Mandarin (4%, 11/252). When analyzed separately by campus, Arabic was reported by four Peoria students (9% of 45 respondents), making it the second most common non-English language reported on that campus, and Urdu was as common as Mandarin in the Rockford campus (each reported by 2/44 respondents, tying for third most common language reported).

### Students’ prior language-related experiences in healthcare

Students reported experiences in three specific categories: (1) interactions with linguistically diverse patients, (2) assessment of non-English language skills, and (3) any prior training related to working with medical interpreters.

### Interactions with linguistically diverse patients

More than half of session one respondents reported previous experience providing medical care to patients with NELP (55%, 138/252), which was true across respondents who were multilingual as well as those who were monolingual English-speaking: 56% (104/186) of multilingual students and 53% (34/64) of monolingual students reported previously caring for this population (*p* = .699) and regardless of campus (*p* = .095).

We asked students to select and describe the nature of their prior interactions with NELP patients. The largest subset (28%, 62/224) had been asked to serve as interpreters, of whom ten (5% of 224) reported having received any training on how to interpret and none had been certified. The majority who reported serving as ad hoc interpreters indicated playing this role with their own family members, and some in clinic/hospital settings when volunteering or shadowing or when working in other healthcare jobs prior to medical school.

When analyzing associated free-text responses, we identified six themes (Table 2). Most (113/172, 66%) described experiences an observer in the care of patients with NELP. Thirty-four students (20% of 172) elaborated on their experiences as ad hoc interpreters. For example, one described that “Growing up I had to often translate for my mother whenever she took us to the pediatrician or when she needed to see her PCP.” A few (22/72, 13%) described the direct care of patients with NELP, and three students had done so as part of research working with patients enrolled in clinical trials.

**Table 2:** Summary of qualitative themes extracted from pre-clinical medical student responses about their prior experiences with non-english language patients (*n* = 172)

Theme	Illustrative quote
Providing direct patient care in non-English languages	“As a medical assistant and ophthalmic technician, I have worked with many patients whose preferred or only language is Spanish. I am generally able to gather a basic ophthalmology HPI in Spanish.”
Observing patient care in populations with NELP	“When I worked as a scribe in the emergency room […] there were a lot of patients that did not have English as their first language.”
Serving as ad hoc interpreter when shadowing/volunteering	“When I shadowed, I often put my language skills into action when resident or attending needed to ask a question that patient did not understand.”
Serving as ad hoc interpreter when accompanying family members	“I often take my grandmother, who only speaks Polish, to her doctors’ appointments.”
Research interactions with NELP populations as human subjects	“I worked as a clinical research assistant prior to medical school and sometimes would interact with patients through a phone interpreter.”

*Abbreviations* NELP, Non-English Language Preference

#### Prior experiences assessing non-english language skills

In session two, we asked students to report whether they had ever been assessed for their non-English language skills in healthcare and whether they had been taught self-assessment techniques. Two students reported having had medically contextualized language assessment; one described having been interviewed to confirm language skills in Spanish prior to volunteering as an assistant at a clinic and another as a community health educator. Twenty-six students (16% of 158) reported having taken and passed some form of general (non-medical) language assessment; most of these respondents described advanced placement language courses or exams in high school or minors/certificates in college (e.g., Spanish, Chinese, Japanese, French, and Korean). Very few (3/158, 2%) reported being exposed to any language tools for progressive self-assessment.

#### Prior training related to working with medical interpreters

In session three, over half of the respondents (144/224) reported previous exposure to patient care mediated by a professional medical interpreter. Most reported having received partial training to work with interpreters (153/224, 68%), but when invited to elaborate through free-text responses, many of the respondents (26/54, 48%) explained that they were referring to our preceding language sessions given as part of this language equity curriculum. One student explained, “Previous sessions have discussed the importance of using a medical interpreter to communicate and understand the nuances of explanations and to prevent missing vital details in patient care.” Other training themes identified included self-study opportunities to learn these skills (6/54, 11%) and partial training in working with interpreters as part of research experiences (1/54, 2%).

### Post-session change in knowledge and attitudes

Across the three sessions, student knowledge and attitudes improved for most questionnaire items (Table 3). In the first session, students reported increased confidence in explaining the role of language in health and in addressing language issues that may arise in healthcare settings (*p* < .001). Students were less likely to indicate that “language is a barrier” to quality medical care (*p* < .001; *p* = .015 for unpaired and paired responses, respectively), suggesting that the session helped some students gain a more nuanced understanding of language as an opportunity to improve quality medical care rather than as a barrier. However, after the first session, students were more likely to respond *incorrectly* to the definition of medical interpreter (*p* = .003 [unpaired]; *p* < .001 [paired]) and to report discomfort with the prospect of mixing languages during communication with patients (*p* = .010 [unpaired]; *p* = .004 [paired]), suggesting that one session about language and healthcare alone is insufficient at gaining the necessary skills to provide language-appropriate care.

**Table 3:** Changes in learner knowledge and attitudes pre- and post-session for each session in the language equity pre-clinical curriculum

Session	Questionnaire Item	Unpaired Data*			Paired Data**		
		Pre-Session	Post-Session	*p*-value	Pre-Session	Post-Session	*p*-value
**Session 1**: **Role and Impact of Language and Health** Pre: *n* = 252, Post: *n* = 117	Language is a barrier to quality medical care.	99%	85%	< 0.001	99%	85%	0.015
	Language concordance improves patient outcomes.	99%	99%	0.741	99%	99%	0.926
	Professional interpreters eliminate language discordance in medical encounters.	60%	76%	0.003	65%	76%	< 0.001
	It is appropriate to use mixed languages when communicating with patients.	68%	55%	0.010	70%	55%	0.004
	I can accurately and confidently explain the role and impact of language in health.	71%	100%	< 0.001	72%	100%	< 0.001
	Knowledge: Patient presents for medical care but does not speak the same language as the clinician	86%	95%	0.037	87%	95%	< 0.001
	Confidence and preparedness to address language issues that may arise in health care with linguistic minorities	62%	95%	0.067	63%	95%	0.111
**Session 2**: **Physician Language Proficiency Standards** Pre: *n* = 158, Post: *n* = 67	Accurately and confidently define what is meant by “false fluency.”	43%	100%	< 0.001	49%	100%	< 0.001
	Accurately and confidently define what is meant by “medical language proficiency”	65%	100%	< 0.001	71%	100%	< 0.001
	Taking a formal medical language exam would replace the need for self-assessing language skills	17%	9%	0.111	16%	9%	0.230
	The better a medical student/doctor’s language skills are the more accurate their self-assessment will be	56%	28%	< 0.001	48%	28%	0.002
	I have a plan for progressive self-assessment of my non-English language skills	24%	91%	< 0.001	16%	91%	< 0.001
	I feel confident in discussing appropriate use of non-English medical language skills with other students	51%	97%	< 0.001	52%	97%	< 0.001
	I believe my current skills in any non-English language would enable me to communicate directly with patients (without an interpreter) to fulfill my clinical responsibilities	16%	18%	0.808	20%	18%	0.651
**Session 3: Working Effectively with Medical Interpreters** Pre: *n* = 227, Post: *n* = 79	Accurately and confidently explain the role and impact of professional medical interpreters in healthcare	88%	97%	0.016	92%	97%	0.147
	Professional interpreters eliminate language discordance in medical encounters	74%	81%	0.239	70%	81%	0.097
	Confident in my ability to perform my patient care duties while working with a professional medical interpreter	69%	92%	< 0.001	66%	92%	< 0.001
	Confident in how to best work with onsite professional medical interpreters	54%	96%	< 0.001	48%	96%	< 0.001
	Confident in how to best work with telephone professional medical interpreters	44%	87%	< 0.001	38%	87%	< 0.001
	Confident in how to best work with video professional medical interpreters	42%	90%	< 0.001	43%	90%	< 0.001
	Confident in my ability to recognize potential pitfalls and/or errors that can occur during a healthcare encounter involving medical interpretation	51%	97%	< 0.001	54%	97%	< 0.001
	Accurately and confidently explain the medical interpreter policy at my clinical work site	29%	86%	< 0.001	29%	86%	< 0.001
	Confident in my ability to advocate for requesting a professional medical interpreter when needed	79%	95%	0.001	84%	95%	0.021
	Confident in my ability to complete the logistical process of requesting a professional medical interpreter	26%	84%	< 0.001	19%	84%	< 0.001

Note: Percentages shown represent the respondents who indicated “Agree” or “Somewhat Comfortable” and above to pre- and post-questionnaire items. *P*-values based on chi-squared test

*Unpaired data include responses from all student participants, regardless of whether they completed both the pre and post-questionnaire

**Paired data only include responses from student participants who completed both the pre and post-questionnaire. Sample sizes for paired data are as follows: session 1, *n* = 117; session 2, *n* = 67; session 3, *n* = 79

Following session two, students were more likely to correctly define “false fluency” and “medical language proficiency” and describe the accuracy limitations of language self-assessment (all *p* < .001). They were also more confident in discussing language skills with peers. Although more than 75% of pre-session respondents reported having skills in at least one non-English language, only 16% reported a self-perceived ability to use non-English language skills with patients without an interpreter, and this percentage did not significantly change after the session (18%, *p* = .808[unpaired]; *p* = .651 [paired]).

After the final session, when looking at the full data set (unpaired responses), students were more likely to successfully define the role and value of medical interpreters following the session (*p* = .016), though when comparing paired data only, the difference was not significant (*p* = .147) as the number of students that had this knowledge prior to the session was high. This was the only item that differed in statistical significance when examining unpaired versus paired data. Importantly, students were more likely to recognize common pitfalls during interpreter-mediated encounters (*p* < .001). They also reported increased confidence in the logistical process of requesting a medical interpreter at their clinical sites, as well as the steps for collaborating with a professional medical interpreter via in person, through video, and via phone (all *p* < .001).

### Intent to apply concepts learned

After each session, students had the option to provide free-text responses to describe the ways they plan to apply the new knowledge into their practice. One-hundred and two students provided such responses for session one (87% of 117 post-session respondents), 62 for session two (93% of 67 post-session respondents), and 56 students for session three (71% of 79 post-session respondents). Table 4 summarizes the themes identified across responses.

**Table 4:** How students plan to apply language-appropriate healthcare knowledge: themes identified in qualitative analysis of post-session responses

Session	Theme	Number of respondents (%)
**Session 1: Role and Impact of Language and Health** *n =* 102	Plan to increase patient advocacy	29 (28%)
	Plan to increase professional interpreter use	29 (28%)
	Plan to gain/improve knowledge of language resources/policies	17 (17%)
	Plan to be more aware of impact of language discordance	16 (16%)
	Plan to gain/improve skills in a non-English language	7 (7%)
	Plan to increase awareness of biases about non-English speakers	4 (4%)
**Session 2: Physician Language Proficiency Standards** *n* = 62	Plan to openly discuss medical language skills, uncertainties, or questions with peers or supervisors	39 (63%)
	Plan to schedule time for self-assessment	35 (56%)
	Plan to take a medical language course	27 (44%)
	Plan to take a formal assessment exam	26 (42%)
**Session 3: Working Effectively with Medical Interpreters** *n* = 56	Plan to gain/improve knowledge of language resources/policies	24 (36%)
	Plan to increase patient advocacy	15 (36%)
	Plan to increase professional medical interpreter use	10(14%)
	Plan to adopt more collaborative approach when interacting with professional interpreters	7 (14%)

Following session one, the most commonly recurring themes for skills application were focused on advocacy and systems-based practice with 29 students (28% of 102) sharing their plans to advocate for patients’ language rights and 29 (28% of 102) planning to increase their use of professional interpreters: “I plan to always make it a top priority for the patient to have interpretive resources and for us both to be able to fully understand one another.”

After session two, more students reported a plan for progressive non-English language self-assessment than before the session (91% of post-session respondents [61/67] v. 24% of pre-session respondents [38/158], *p* < .001). Most post-session respondents indicated a plan to pursue multiple approaches to advance or assess their language skills (61%, 38/62), including taking a language course (44%, 27/62), taking a formal assessment exam (42%, 26/62), scheduling time for progressive self-assessment using the validated tools provided during the session (56%, 35/62), and discussing medical language skills, uncertainties, or questions with peers and supervisors (63%, 39/62). Students could use a free-text box elaborate on ways they plan to apply what they learned, and 54 did so. From those responses, we identified two major themes: most discussed how they will assess their second language skills (61%, 33/54) and others wrote about increasing their recognition of false fluency (39%, 21/54). One participant described this knowledge gain about false fluency as being aware that “*perceived* language skills are different than *actual* skills.”

Following session three, we identified four themes across free-text responses about knowledge application. Twenty-four students (43% of 56) referenced their increased practical knowledge about hospital policies regarding language use and how they will use that information to better access professional interpreters in the future. One student shared, “I feel more confident in understanding the process of requesting and utilizing an interpreter, making it more likely that I will advocate for their use whenever needed.”

### Student feedback and curricular updates

Most students consistently reported that the didactic components were useful in helping them achieve the session learning objectives (94% [110/117], 100% [64/64], and 85% [67/79] for each session, respectively). Respondents rated the interactive elements similarly positively with regards to usefulness toward achieving learning objectives (90% [105/117], 90% [58/64], and 86% [68/79] for each session, respectively). When asked about feedback for future improvements, almost half of session one respondents suggested to shorten the breakout session duration (44%, 44/99); this informed our planning for the second session. Some students proposed expanding the scope to include more facilitator examples of lived experiences working with NELP populations, as well as education on nonverbal communication strategies (25%, 25/99). After session two, students suggested topics that could be explored for additional content (29%, 14/48), such as information about available resources for learning a non-English language for medical use. Following the third session, the most common opportunity for improvement noted by students was to the desire for hands-on practice experience in working with medical interpreters (43%, 31/72).

Based on this feedback, following the successful implementation of the three-part language equity series, the medical school implemented several curricular updates. First, a fourth educational intervention was developed during a required course that takes place after the first few months of clinical clerkships. This fourth session was added to enable students to reflect about language-appropriate care in the context of patient safety following their initial clinical experiences. Second, the medical school improved the accessibility of language services information to students in the clinical years by creating an informational tag that could be attached to student identification badges. Third, the coalition has begun working with the institution’s simulation center on recruitment of a linguistically diverse pool of standardized patients. This is a first step in planning for standardized patient encounters where formative and summative evaluation of students’ language-appropriate communication can be more broadly incorporated.

## Discussion

We developed a longitudinal language equity curriculum to equip medical students with foundational language equity concepts and skills. One of the strengths of our curriculum is its implementation across urban, suburban, and rural sites. Over half of respondents indicated past exposure to care of NELP patients regardless of their own non-English language skills. This finding supports the need for language equity education for all students, not just those whose skills are proficient enough to provide language-concordant care nor those who are internally motivated to sign up for language electives or extracurricular experiences. Notably, by the third session, both multilingual and monolingual students across all three campuses described a plan to advocate on behalf of their patients with NELP if they observed poor communication practices. Similarly, many students planned on increasing their own use of professional interpreters.

By collecting language skills data, our intervention facilitated recognition of the rich linguistic diversity of the student body in participating schools; three quarters of respondents reported skills in at least one non-English language. Language skills are invisible characteristics that may intersect with other elements of diversity such as race, ethnicity, immigration story, and nationality, among others [40, 41]. While many institutions offer some form of medical Spanish education [24], and Spanish is the language of greatest need in most areas of the US, [1, 2] the linguistic diversity of our study’s participants highlights the need for preparing all learners (not just Spanish speakers) to appropriately use their language skills clinically. Students with skills in less common languages may have difficulty identifying educational resources for advancing or assessing their proficiency, making language equity education an important and foundational way to engage students in language-appropriate care regardless of languages spoken. Institutionalizing language equity education is an opportunity to highlight language – an understudied facet of student diversity – and engage learners and faculty in active discussions in which multilingual experiences are explicitly valued. Embedding language equity concepts, including skills for working with interpreters [42] as part of core, required clinical skills training sends an important message that skills for language-appropriate care are a key part of a comprehensive toolbox for all physicians.

Students learned the importance of progressively self-assessing their skills in languages besides English to accurately determine when they should partner with a qualified medical interpreter. For monolingual English-speaking students or multilingual students with intermediate or lower skill levels in a language, they should always partner with an interpreter via remote or in-person modes [10]. The choice of mode of interpretation depends on availability (which can vary by clinical site) and encounter complexity. Students received a list of their local campus’ clinical sites and information on how to access language services at each site. For multilingual students with advanced or higher skills in a language, proficiency testing is recommended to certify their skills; additionally, self-assessment should be continually applied since some clinical situations might pose unexpected or complex linguistic challenges with which even an advanced speaker may need additional language support [35].

This study sheds light on persistent structural barriers that disproportionately affect multilingual trainees and contribute to language-related health disparities for patients [43]. A concerning number of students reported having been asked to serve as ad hoc interpreters. These findings are consistent with prior literature about ad hoc interpreting by trainees, [14, 44] and support the need to revisit hospital policies and training for all healthcare staff (e.g., resident and attending physicians, nurses, etc.) who may be unaware of language-related legal requirements or best practices. Importantly, our longitudinal intervention resulted in many respondents developing a plan to apply strategies to improve and/or assess their language skills and to openly discuss language issues with peers, staff, or supervisors. Future research should explore long-term outcomes by evaluating the rates of working with interpreters, participating in language courses, or taking language assessment examinations for students exposed to the foundational pre-clinical language equity curriculum.

Our study had some limitations. While overall student engagement in the course was excellent, we observed attrition in the number of students who completed the questionnaires, with 25% of participants completing the final session’s post-questionnaire, potentially resulting in sampling bias. Also, a small subset of students (17%) participated in the course but did not complete the questionnaires. Since language data is not routinely collected, we have no way of knowing whether this subset of students differed from respondents with regards to their linguistic profile. All students were from the same institution despite being situated on three different campuses with unique patient populations. Student feedback prompted some course improvements, such as creating a role-play video to illustrate an example of an ethical dilemma that may arise during a medical encounter between a patient with NELP and a partially fluent medical student. In future courses, the impact of these changes should be evaluated. Secondly, our primary outcomes were self-perceived attitudes, confidence, and intent to apply concepts learned; it would be important to correlate these findings with students’ performance on experiential opportunities, such as standardized patient encounters, to assess the clinical skills taught in the course and receive formative feedback. Moreover, it would be valuable to track learners’ progress throughout the clinical years of medical school and residency through metrics such as interpreter utilization, periodic language proficiency assessments, clinical outcomes, and patient satisfaction.

## Conclusions

Incorporating a pre-clinical undergraduate medical curriculum is a strategy for exposing all medical students to foundational education about improving health equity through language-appropriate care. Next steps should include exploring methods for evaluating these skills, including students’ communication with linguistically diverse populations and interprofessional collaboration with medical interpreters during clinical clerkships. Future research should also consider the potential indirect impact on language services utilization by other members of the healthcare team who might learn about language-appropriate care from medical students who took the course and explore whether language equity education improves belonging for students and clinicians from linguistically diverse backgrounds.

## Data Availability

Data is provided within the manuscript; additional data requests should be directed to the corresponding author.

## Abbreviations

DoCS: Doctoring and Clinical Skills
LEP: Limited English Proficiency
NELP: Non-English Language Preference
UICOM: University of Illinois College of Medicine
